# Early Nutrition and Weight Gain in Preterm Newborns and the Risk of Retinopathy of Prematurity

**DOI:** 10.1371/journal.pone.0064325

**Published:** 2013-05-29

**Authors:** Deborah K. VanderVeen, Camilia R. Martin, Reshma Mehendale, Elizabeth N. Allred, Olaf Dammann, Alan Leviton, for the ELGAN Study Investigators

**Affiliations:** 1 Department of Ophthalmology, Boston Children’s Hospital, Harvard Medical School, Boston, Massachusetts, United States of America; 2 Department of Neonatology, Beth Israel Deaconess Medical Center, Harvard Medical School, Boston, Massachusetts, United States of America; 3 Neuroepidemiology Unit, Boston Children’s Hospital, Boston, Massachusetts, United States of America; 4 Department of Neurology, Harvard Medical School, Boston, Massachusetts, United States of America; 5 Department of Biostatistics, Harvard School of Public Health, Boston, Massachusetts, United States of America; 6 Department of Public Health and Community Medicine, Tufts University School of Medicine, Boston, Massachusetts, United States of America; 7 Perinatal Neuroepidemiology Unit, Hannover Medical School, Hannover, Germany; Massachusetts Eye & Ear Infirmary, Harvard Medical School, United States of America

## Abstract

**Objective:**

To identify nutritional and weight gain limitations associated with retinopathy of prematurity (ROP) severity among very preterm newborns.

**Patients and Methods:**

1180 infants <28 weeks GA at birth with ROP examination results were grouped and analyzed by quartile of weekly total calorie, carbohydrate, protein, and lipid intake, as well as growth velocity between postnatal days 7 and 28 (adjusted for GA and birth weight Z-score). ROP was categorized by development of no, mild (<prethreshold), type 2, or type 1 ROP, as well as markers of ROP severity including stage 3 ROP, zone 1 disease, and plus disease. Associations between nutritional intake and ROP severity were compared.

**Results:**

Greater risk for Type 1 ROP (risk/95% confidence intervals) was found for infants with lowest quartile receipt of lipids (2.1/1.1, 3.8), total calories (2.2/1.4, 3.6), and carbohydrates (1.7/1.1, 2.9). Development of zone 1 ROP was associated with lipid or total calorie intake in the lowest quartile, and development of stage 3 ROP was associated with lowest quartile of total calorie intake. Growth velocity in the lowest quartile was associated with increased risk of any ROP, including type 1 ROP.

**Conclusion:**

The risk of developing severe ROP in extremely premature infants might be reduced by improving nutritional support, specifically targeting lipids and total calories, and perhaps by improving weight gain.

## Introduction

Retinopathy of prematurity (ROP), a neovascular disease of the retina found in very premature infants, can result in life-long visual limitations. Identifying the antecedents of ROP provides additional information on relevant pathogenic mechanisms involved and has the potential to help reduce the occurrence of this disease and its consequences. [1].

Early nutritional support is a critical part of optimizing growth and development in the premature infant, including normal retinal vascularization. Insulin-like growth factor (IGF)-1 is important for normal infant growth [2]–[4] and retinal vascularization, [5]–[8] and is regulated by total caloric and protein intake. [2] Lipid emulsions are an important part of nutritional support; omega-3 long chain polyunsaturated fatty acids have been shown to suppress inflammatory factors and contribute to antiangiogenic and neuroprotective mechanisms in the retina. [9]–[13] Early weight gain is an important marker of nutritional status, and poor postnatal weight gain has been associated with an increased risk of severe ROP. [14]–[17] Algorithms have been developed that employ postnatal weight gain and IGF levels to predict development of severe ROP. [18], [19].

Premature infants enrolled in the ELGAN (*e*xtremely *l*ow *g*estational *a*ge *n*ewborn) Study had assessments of nutritional support (carbohydrate, protein, lipids, and total calories) and weight measurements during the first 28 days of life. We evaluated the relationships between components of early nutritional support and weight gain with markers of ROP severity in this at-risk population.

## Patients and Methods

### Sample

The ELGAN Study, a prospective cohort study designed to identify characteristics and exposures that increase the risk of structural and functional neurologic disorders, enrolled 1506 extremely low gestational age newborns (birth between 23 and 27 6/7 weeks of gestation) at one of 14 participating institutions between 2002–2004. [20] The enrollment and consent processes were approved by the individual institutional review boards, and research conducted according to the principles expressed in the Declaration of Helsinki. Written informed consent was obtained from the parents and/or legal guardians of all infants enrolled in the study.

Of these newborns, 1187 had their weight recorded on days 7 and 28, as well as nutrition information collected on days selected for analysis (days 3, 7, 14, and 21). The gestational age, birth weight, and other perinatal characteristics of 1064 of these 1187 infants are presented elsewhere. [21] Results of retinal examinations were available for 1180 of these infants. The enrollment and consent processes were approved by the individual institutional review boards.

### Infant Characteristics

Gestational age (GA) estimates were based on a hierarchy of the quality of available information with estimates based on the dates of embryo retrieval or intrauterine insemination or fetal ultrasound before the 14^th^ week of gestation (62%) as the most desirable. Next most desirable in sequential order were estimates based on a fetal ultrasound at 14 or more weeks of gestation (29%), last menstrual period without fetal ultrasound (7%), and GA recorded in the log of the NICU (1%).

### Nutrition Data

Detailed nutritional data were collected daily for the first 7 postnatal days, then weekly on days 14, 21, and 28. [22] Type and volume of intravenous solutions (including composition of parenteral nutrition) as well as type and volume of enteral feedings (including caloric additives) were routinely recorded. All nutritional practice variables summarize nutritional intake from both parenteral and enteral routes.

Nutrient information was calculated for the macronutrients protein, carbohydrates, and fats. These totals were calculated by multiplying the daily total volume of the diet by the macronutrient content as specific on the nutritional label for all formula types and nutritional additives, such as powdered proteins or fat oils. For breast milk, we calculated nutritional intake using values for mature milk (Ross Neonova system, version 4.5, 1999) since by 14 days the composition of preterm milk and term milk are similar. Although human milk composition can be very dynamic, it was not feasible in this study to capture the composition of every breast milk sample provided to the infants.

Total kilocalories per day were divided by the birth weight until the birth weight was surpassed, then by the weight on the day the nutritional information was collected. The final units for the macronutrient calculations were kcals/kg/day. For analysis, we categorized the mean total nutrient (g/kg/day) and mean total calories (kcal/kg/day) on each of four selected days (3, 7, 14 and 21) into quartiles.

### Weight Change

Body weight was measured each day in the first postnatal week, then on days 14, 21 and 28. The rate in growth is expressed as g/kg/day. Because ELGANs tend to lose weight during the first postnatal week [23] our measure of weight gain was calculated for the interval between days 7 and 28 [((wt28-wt7)/wt7)/(28-7) 1000×g/kg/day]. We categorized this measure of weight gain into quartiles.

### Eye Examinations

Retinal examinations were performed by ophthalmologists experienced in ROP screening. Participating ophthalmologists helped prepare a manual and data collection form, and then participated in efforts to minimize observer variability. Definitions of terms were those accepted by the International Committee for Classification of ROP. [24] In keeping with guidelines, [25]–[26] the first ophthalmologic examination was within the 31^st^ to 33^rd^ post-menstrual week. Follow-up exams were as clinically indicated until normal vascularization began in zone III.

For purposes of discussion, prethreshold ROP is defined as any ROP in zone 1, stage 2 ROP in zone 2 with plus disease, or stage 3 ROP in zone 2 with or without plus disease. Prethreshold ROP is designated as type 1 or type 2 ROP, [27] as follows:

Type 1 ROP:

- zone I, any stage ROP with plus disease;
- zone I, stage 3 ROP without plus disease; or
- zone II, stage 2 or 3 ROP with plus disease.

Type 2 ROP

- zone I, stage 1 or 2 ROP without plus disease or
- zone II, stage 3 ROP without plus disease.

During the study period, criteria for treatment of ROP varied, due to participation in the Early Treatment for Retinopathy of Prematurity (ETROP) randomized trial, or due to subsequent changes in treatment recommendations [27]–[28]. We did not evaluate need to treat as a separate indicator of severity, since some eyes of infants in the ELGAN study received treatment for high-risk prethreshold or type 1 ROP, while others received treatment only when threshold ROP developed. Threshold ROP was defined as 5 contiguous or 8 cumulative clock hours of stage 3 ROP with plus disease in zone 1 or 2, so all cases previously designated as threshold or near threshold ROP are included in the current classification of type 1 ROP.

In this analysis, we categorized ROP severity in four ways: no ROP, mild ROP (<prethreshold), type 2 ROP, and type 1 ROP. Additional markers of severity were noted as≥stage 3 ROP, zone I disease, and presence of plus disease. The comparison groups in these analyses include infants with no or mild ROP.

### Statistical Analyses

We evaluated two hypotheses. First, infants who received nutrition (*i.e*., protein, carbohydrate, fat and total calories) in the lowest quartile are not at increased risk of any measure of ROP severity. Second, infants in the lowest quartile of weight gain are not at increased risk of any indicator of ROP severity.

We evaluated these hypotheses in two ways. One set of analyses was characterized by logistic regression models of each level of severity compared to the referent group of children who did not have any ROP. To account for the possibility that infants born at a particular hospital are more like each other than like infants born at other hospitals, a hospital cluster term was included in all of these models. [29].

Because each level of severity is mutually exclusive and each is appropriately compared to the same referent group of infants without any ROP, we also created multinomial (aka polytomous or polychotomous) logistic regression models. [30], [31] Multinomial models do not allow a cluster term to be included.

All models adjusted for gestational age category (23–24, 25–26, 27 weeks), and birth weight Z-score category (<−2, ≥ −2 to<−1, ≥ −1 to 0, ≥0). We calculated odds ratios (and 95% confidence intervals) with the aid of the logistic regression models.

By and large, the two sets of analyses provided similar findings. For economy of space we present only results obtained with the multinomial models.

## Results

Of the 1180 infants studied, 876 (74%) developed ROP (Table 1). Thirty percent (359/1180) developed prethreshold ROP, and 13% (156/1180) developed Type 1 ROP. ROP≥ stage 3 developed in 345 infants; zone 1 disease was present in 89 infants.

**Table 1:** Sample description.

		Yes	No
Enrolled		1506	
Weight measured: on days 7 and 28		1187	319
Nutrition information available*		1187	0
ROP diagnoses available		1180	7
No ROP (this is the referent group)		304	
Therapy	Mild	517	
oriented	Type 2	203	
	Type 1	156	
Stage	1	260	
	2	271	
	≥3	345	
Zone	III	52	
	II	765	
	I	89	
Plus disease	Yes	131	

* collected on selected days: 3, 7, 14, and 21.

**Figure 1:**
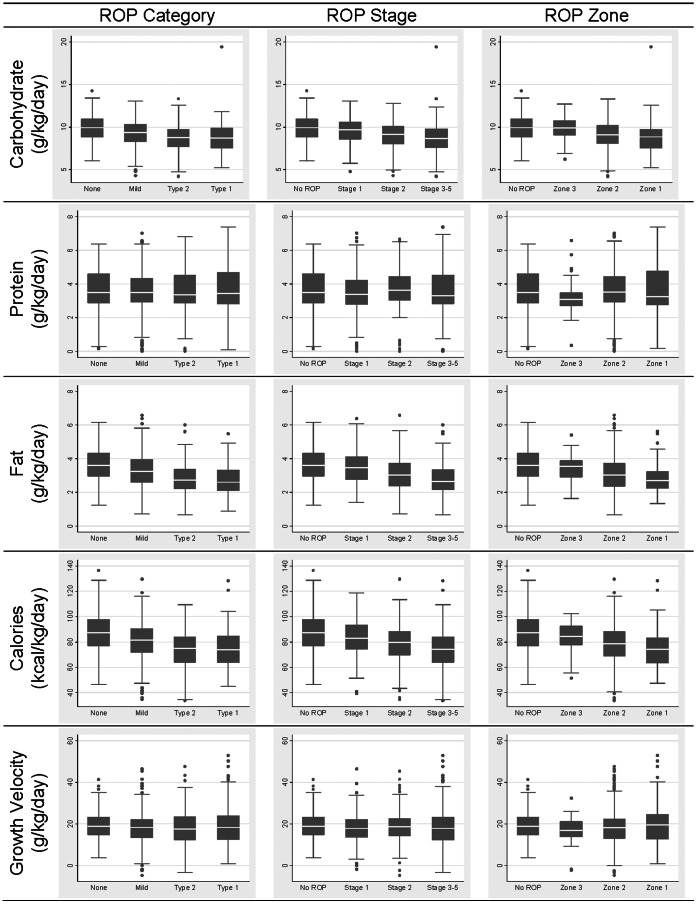
Box and whiskers displays of total carbohydrate, protein, and fat (g/kg/day), total calories (kcal/kg/day), and growth velocity (g/kg/day) among children classified by ROP severity.

Figure 1 shows the distribution of mean total nutrients and calories received on days of life 3, 7, 14 and 21. Not only was the median amount of carbohydrates received by children who had no ROP higher than those who developed ROP, but the more severe the ROP, the lower the median. A similar pattern was seen for median total fat, and mean total calories, but not for median total protein. In contrast to the linear relationships between the levels of some nutrients and ROP severity, no such pattern was seen between weight gain between days 7 and 28 and ROP severity.

Table 2 displays the risk of ROP for infants whose receipt of each nutrient or calorie intake was in the lowest quartile, adjusted for gestational age and birth weight Z-score. Increased risk of type 1 ROP was found for infants in the lowest quartile for carbohydrate, fat, and total calorie intake. Increased risk of≥stage 3 ROP was found for infants in the lowest quartile of total calorie intake, and increased risk of zone 1 ROP was found for infants in the lowest quartile of fat or total calorie intake. Receipt of low amounts of protein was not associated with appreciably increased risk of any ROP severity.

**Table 2:** The risks (and 95% confidence intervals) of the ROP level identified on the left among infants whose receipt of the nutrient or calories at the top of each column was in the lowest quartile relative to the risk among children whose receipt was in the top 3 quartiles.

Retinopathyof prematurity		Lowest quartile			
		CHO	Protein	Fat	Calories
Therapy	Mild	1.5 (0.99, 2.2)	0.9 (0.6, 1.2)	**1.7 (1.1, 2.6)**	1.0 (0.7, 1.5)
oriented	Type 2	**1.9 (1.2, 3.0)**	0.9 (0.6, 1.4)	**2.9 (1.8, 4.8)**	**1.7 (1.1, 2.7)**
	Type 1	**1.7 (1.1, 2.9)**	1.1 (0.7, 1.8)	**2.8 (1.7, 4.7)**	**2.2 (1.4, 3.6)**
Stage	1	1.1 (0.7, 1.8)	1.1 (0.8, 1.6)	1.1 (0.7, 1.9)	0.8 (0.5, 1.3)
	2	**1.8 (1.1, 2.7)**	0.7 (0.4, 1.00)	**2.3 (1.4, 3.6)**	1.2 (0.8, 1.9)
	≥3	2.0 (0.3, 3.1)	1.0 (0.7, 1.5)	3.2 (2.0, 5.0)	**2.1 (1.4, 3.2)**
Zone	III	1.3 (0.6, 2.8)	1.8 (0.9, 3.3)	1.1 (0.5, 2.9)	1.1 (0.5, 2.4)
	II	**1.6 (1.1, 2.4)**	0.8 (0.6, 1.1)	**2.1 (1.4, 3.2)**	1.3 (0.9, 1.8)
	I	1.8 (0.99, 3.2)	1.4 (0.8, 2.5)	**2.6 (1.4, 4.6)**	**2.2 (1.2, 3.8)**
Plus	Yes	1.1 (0.8, 1.7)	1.2 (0.8, 1.8)	1.4 (0.9, 2.0)	1.5 (0.98, 2.2)

These have been adjusted for gestational age and birth weight Z-score. **Bolded** odds ratios are significant at the 0.05 level.

Unlike Table 2, Table 3 shows the three lower quartiles for growth velocity, which provided an opportunity to see if a dose-response characterized the findings. Weight gain in the lowest quartile was associated with increased risk of all stages of ROP, including type 1ROP. Less limited weight gains were not associated with increased risk of any severity level of ROP.

**Table 3:** The risks (and 95% confidence intervals) of the ROP level identified on the left among infants classified by the quartile of their weight gain between days 7 and 28 relative to the risk among children whose weight gain was in the highest quartile.

		Growth velocity quartile		
Retinopathy of prematurity		Lowest	Low middle	Hi middle
Therapy	Mild	**1.9 (1.2, 3.0)**	1.3 (0.8, 1.9)	1.3 (0.9, 2.0)
oriented	Type 2	**2.0 (1.2, 3.5)**	1.2 (0.7, 2.0)	0.9 (0.5, 1.6)
	Type 1	**1.9 (1.1, 3.5)**	0.8 (0.5, 1.5)	0.8 (0.5, 1.5)
Stage	1	**1.9 (1.1, 3.1)**	1.3 (0.8, 2.1)	1.2 (0.8, 1.9)
	2	**1.8 (1.1, 3.0)**	1.2 (0.7, 1.9)	1.3 (0.8, 2.2)
	≥3	**2.2 (1.4, 3.7)**	1.1 (0.7, 1.7)	0.9 (0.6, 1.5)
Zone	III	2.2 (0.9, 5.7)	2.2 (0.9, 5.2)	1.4 (0.6, 3.6)
	II	**2.0 (1.3, 3.0)**	1.2 (0.8, 1.8)	1.2 (0.8, 1.8)
	I	1.5 (0.8, 3.0)	0.5 (0.2, 1.1)	0.8 (0.4, 1.5)
Plus	Yes	1.1 (0.7, 1.8)	0.8 (05, 1.4)	0.7 (0.4, 1.2)

These have been adjusted for gestational age and birth weight Z-score. **Bolded** odds ratios are significant at the 0.05 level.

Males and females did not differ in nutritional intake, growth velocity, or ROP risk.

## Discussion

In this cohort of extremely low gestational age newborns, intake of total calories, lipids, and carbohydrates in the lowest quartile, measured on postnatal days 3,7,14, and 21, was associated with increased risk of type 1 ROP. Similarly, infants with growth velocity in the lowest quartile were at higher risk of developing any ROP, including type 1 ROP. These findings raise the possibility that improving the early nutrition of these infants may help prevent development of sight-threatening ROP.

In a previous report from the ELGAN Study, the intake of protein and fat were within the generally accepted dietary goals for ELGANs, while the goals for intake of carbohydrate and total calories were not achieved. [22] Controversy continues about what nutritional targets should be in preterm newborns. [32]–[36] While growth velocities were maintained above 15 mg/kg/day, most infants had a weight Z-score on day 28 that was lower than at birth. This might be viewed as an indicator of extra-uterine growth failure. Not surprisingly, infants who received protein, fat, and carbohydrate amounts in the lowest quartile on day 7 were more likely than their peers to have growth velocity in the lowest quartile. We do not know why delivery of these nutrients was suboptimal. While it is possible to observe associations between low nutrition and co-morbidities, determining whether the suboptimal delivery of nutrients contributed to development of ROP or whether co-morbidities caused the poor dietary intake is beyond the scope of information collected for this study.

Receipt of higher amounts of intravenous fat emulsion (2 g/kg/day; Intralipid) during the first week of life has been associated with decreased rates of ROP. [9] Our observation that infants in the lowest quartile for lipid intake were at increased risk of prethreshold ROP supports this finding. The quantity but not quality of lipid intake was recorded, so it is possible that some newborns experienced lipid component deficiencies known to be important for retinal vascular development and stability, such as the omega-3 long chain polyunsaturated fatty acid docosahexaenoic acid (DHA). [12], [37] Parenteral lipid supplements given in the United States do not contain DHA, and the addition of DHA to infant formula commenced near the beginning of this study period (2002). Even those who received breast milk early did not have amounts recorded or DHA concentration analyzed.

Poor postnatal weight gain is a predictor of severe ROP. [14]–[19], [38]–[41] The WINROP algorithm, which measures a cumulative slowdown in weight gain, has a high sensitivity (90–100%) for detecting infants at risk of developing prethreshold ROP. A caveat to assessment of weight gain alone is the need to distinguish those infants who have non-physiologic weight gain, such as weight gain from body edema, hydrocephalus, or other causes. Infants who develop proliferative ROP often have co-morbidities such as bronchopulmonary dysplasia and necrotizing enterocolitis, which might limit nutrition and weight gain. In light of the associations between IGF-1 blood concentrations and weight gain as well as ROP risk [5]–[8], [18] it is possible that low weight gain is not in the causal chain leading to ROP, but instead is merely an indicator of IGF-1 concentration, which might be in the causal chain. While IGF-1 concentration is unlikely to influence administration of nutrients, it may influence utilization of existing nutrients.

Although many studies have reported low weight gain as a risk factor for ROP (Table 4), and one clinical trial has assessed the contribution of intravenous fat emulsions to reducing ROP risk, this is the first observational study of suboptimal early nutrition as a risk factor for indicator of ROP severity in ELGANs. We found infants receiving the lowest quartile of lipid, carbohydrate, and total calories to be at increased risk of type 1 ROP. Higher amounts of lipid emulsion in the early days of postnatal life, in addition to DHA supplementation, could theoretically reduce ROP risk. Since higher amounts of IV lipid appear to be well tolerated, and given the importance of DHA in retina and brain development [42]–[46], this seems to be an important nutritional component to target. Additionally, maximizing total caloric intake to support early growth appears to show promise of reducing risk of prethreshold ROP.

**Table 4:** Characteristics of the eight studies that evaluated low weight gain as a risk factor for ROP.

Weight gain study	#infants	Entrycriteria	Weight analysis	ROP severity	Other risk factors^∧^
Wallace et al. J AAPOS 2000	111	<30 weeks	Relative weight gain(g/kg/d) through 6 weeks	> stage 3	RBC volume transfused; bacteremia
Allegaert et al. J AAPOS 2003	37	<35 weeks or <2000 g	Absolute weight gain(g/d) through 6 weeks	Threshold	SGA, BW <25%ile
Hellstrom et al. Pediatrics 2009	317	<32 weeks	WINROP*	≥ stage 3	
Fortes Filho et al. Graefes Arch Clin Exp Ophthal 2009	317	<32 weeks or <1500 g	Low weight gain(<51%) at 6 weeks	stage 3 or threshold	
Hard et al. Arch Ophthalmol 2010	366	<32 weeks	WINROP*	≥ stage 3	
Wu et al. 2010 Arch Ophthalmol	318	<32 weeks	WINROP*	≥prethreshold or≥stage 3	
Binenbaum et al. 2011 Pediatrics	367	<1000 g	PINT-ROP**	> stage 3 or threshold	
Wu et al. 2012 Arch Ophthalmol	1706	<32 weeks	WINROP*	Type 1 ROP	
VanderVeen et al.	1180	<28 weeks	Growth velocity between days 7 and 28***	type 2/type 1, ≥stage 3,zone 1, plus disease	Birth weight Z-score; carbohydrate, protein, and lipid intake in lowest quartile

∧Exclusive of birthweight and gestational age at birth.

* Online algorithm that calculates difference between expected “safe” weight gain and actual weight gain; an alarm occurs if the accumulated values exceed a limit.^15,16.^

** Calculated probability of severe ROP based on risk score incorporating GA, BW, and weight gain rate (g/d).

*** [1000×((weight28-weight7)/weight7)/(28-7)] = g/kg/day.

The strengths of this study include the large number of infants studied, enrollment on the basis of well defined gestational age, a protocol with standardized assessments, and prospective collection of nutritional data. Additionally, ROP exams were performed by experienced examiners and ROP data collection occurred in a prospective and standardized fashion. Limitations include less than ideal information about dietary fat components and mode of nutrient delivery.

### Conclusion

Low intake of lipids, carbohydrates, and total calories was associated with increased risks of sight-threatening ROP in this cohort of ELGANs. Together with previous reports that low weight gain is also a risk factor for ROP, this finding suggests that efforts to improve the nutritional intake and weight gain of the most vulnerable newborns might reduce the burden of sight-limiting ROP.

## Supporting Information

File S1 List of all Institutional Review Boards that approved the ELGAN study.
(DOC)
