# Supplementary material for: Early Nutrition and Weight Gain in Preterm Newborns and the Risk of Retinopathy of Prematurity
Source: PLoS One. 2013 May 29;8(5):e64325. doi: 10.1371/journal.pone.0064325 (PMC3667175; doi:10.1371/journal.pone.0064325)
Supplement: File S1 — List of all Institutional Review Boards that approved the ELGAN study. (DOC) [file pone.0064325.s001.doc]

The study was approved at the following institutions:

Institutional Review Board, Human Research Protection Program, Baystate Medical Center, Springfield, MA

Institutional Review Board, Human Subjects Protection Office, Beth Israel Deaconess Medical Center, Boston, MA

The Partners Human Research Committee (Institutional Review Board), Brigham & Women’s Hospital, Boston, MA

The Partners Human Research Committee (Institutional Review Board), Massachusetts General Hospital, Boston, MA

Institutional Review Board, Tufts Medical Center / Tufts University Health Sciences, Floating Hospital for Children at Tufts Medical Center, Boston, MA

Institutional Review Board, University of Massachusetts Medical School, U Mass Memorial Medical Center, Worcester, MA

Human Investigation Committee, Yale University Human Research Protection Program, Yale-New Haven Children's Hospital, New Haven, CT

Forsyth Medical Center Institutional Review Board, Wake Forest University Baptist Medical Center and Forsyth Medical Center, Winston-Salem, NC

Biomedical Institutional Review Board, Office for Human Research Integrity, University Health System of Eastern Carolina, Greenville, NC

Institutional Review Board, Office of Human Research Ethics, North Carolina Children’s Hospital, Chapel Hill, NC

Spectrum Health Institutional Review Board, Helen DeVos Children’s Hospital, Grand Rapids, MI

Institutional Review Board, Sparrow Hospital, Lansing, MI

Chicago Biomedicine Institutional Review Board, University of Chicago Medical Center, Chicago IL

Beaumont Health System Human Investigation Committee, William Beaumont Hospital, Royal Oak, MI

In all instances, written informed consent was obtained from the parents or legal guardians of the infants that participated in the study. The consent procedure was approved by each of the Human Subjects Committees/Institutional Review Boards listed above, and the enrollment, consent processes, and research was conducted according to the principles expresses in the Declaration of Helsinki.
